# Introduction of Medical Physics Leadership Academy (MPLA) case studies

**DOI:** 10.1002/acm2.13187

**Published:** 2021-03-19

**Authors:** Mary Gronberg, Dongxu Wang, Medical Physics Leadership Academy (MPLA) Cases Subcommittee, American Association of Physicists in Medicine (AAPM)

**Affiliations:** ^1^ University of Texas MD Anderson Cancer Center Houston TX USA; ^2^ Memorial Sloan Kettering Cancer Center New York NY USA

Medical physicists are responsible for ensuring the safe and effective use of radiation in medicine. As medical physicists, we are committed to improving patient care through research, clinical practice, and education. We are respected by our healthcare colleagues for our knowledge of radiological physics and technology. Current training programs for medical physicists, mostly in academic healthcare settings, focus on developing technical expertise within the medical physics specialty, while the real workplace involves a multi‐disciplinary healthcare team. A well‐trained, board‐certified medical physicist may not be prepared for the workplace challenges; even an experienced physicist may still face new managerial and interpersonal situations. Medical physics professionalism and leadership, as a collective term, describes a medical physicist's ability to navigate, adapt, and succeed in the complex workplace. Leadership training can help medical physicists develop the necessary skills to navigate their complex environments, improve patient care, and advance our profession.

Many aspects of professionalism and leadership training may be suitable for medical physicists. The American Association of Physicists in Medicine (AAPM) assessed the current leadership needs of its members through a survey and analysis^1^, the Emotional Competency Inventory for Medical Physicists, conducted by Impact International. The survey and analysis included a 360° review of fifty AAPM members. The following leadership skills were identified as weaknesses: conflict management (93% below target), initiative (90% below target), adaptability (73% below target), empathy (60% below target), and emotional self‐awareness (43% below target). The internal AAPM survey and the 360° review highlighted the need for medical physics leadership education.

To address the need for leadership education and development among AAPM members, Medical Physics Leadership Academy (MPLA), a Committee under AAPM's Professional Council, is proposing the use of the case study method. The case study method was started in the 19th century by the Harvard Law School. It has since been adopted by many fields, including medicine. The case study method is based on two components, the case itself and the case discussion. Cases are narratives of challenging situations faced by an individual or group of people that may not have a right or wrong answer. Cases are often real scenarios that people have experienced in the past. Cases contain the background information needed to understand the dilemma; they do not contain analysis or solution. Through the reading, group discussion, or self‐study of the case, the audiences or students analyze the challenging situation and propose action items. They may use role‐play to fully immerse themselves in the case study, for example, in the practice of conflict management. A facilitator may guide the audience or students through the discussion, asking open‐ended questions to facilitate thinking. The facilitator should provide a safe environment and refrain from offering their own opinions.

In this mini‐series, MPLA and MPLA Cases Subcommittee provide the following articles:

1. MPLA Case Writer's Guide^2^
2. MPLA Case 1 and Sample Facilitator's Guide: “Implementing CBCT in a Community Hospital”^3^
3. MPLA Case 2: “A Junior Physicist Attempts to Improve Radiotherapy Workflow”^4^

By developing and utilizing MPLA Case Studies, we hope that medical physicists can learn from each other's experiences and grow as a community in advancing health care.

## Note

^1^
https://w3.aapm.org/leadership/history.php

^2^ [Insert hyperlink or citation for this article, to be published in the same issue of the same journal, as part of a mini‐series.]

^3^ [Insert hyperlink or citation for this article, to be published in the same issue of the same journal, as part of a mini‐series.]

^4^ [Insert hyperlink or citation for this article, to be published in the same issue of the same journal, as part of a mini‐series.]

